# Fatal self-injury in the United States, 1999–2018: Unmasking a national mental health crisis

**DOI:** 10.1016/j.eclinm.2021.100741

**Published:** 2021-02-08

**Authors:** Ian R.H. Rockett, Eric D. Caine, Aniruddha Banerjee, Bina Ali, Ted Miller, Hilary S. Connery, Vijay O. Lulla, Kurt B. Nolte, G. Luke Larkin, Steven Stack, Brian Hendricks, R. Kathryn McHugh, Franklin M.M. White, Shelly F. Greenfield, Amy S.B. Bohnert, Jeralynn S. Cossman, Gail D'Onofrio, Lewis S. Nelson, Paul S. Nestadt, James H. Berry, Haomiao Jia

**Affiliations:** a Department of Epidemiology, West Virginia University School of Public Health, Morgantown, WV, United States; b Department of Psychiatry, University of Rochester Medical Center, Rochester, New York, United States; c Department of Geography, Indiana University-Purdue University at Indianapolis, Indianapolis, Indiana, United States; d Pacific Institute for Research and Evaluation, Calverton, Maryland, United States; e School of Public Health, Curtin University, Perth, Western Australia, Australia; f McLean Hospital, Belmont, Massachusetts, United States; g Department of Psychiatry, Harvard Medical School, Boston, Massachusetts, United States; h Department of Pathology and Radiology, University of New Mexico School of Medicine, Albuquerque, New Mexico, United States; i Northeast Ohio Medical University, Rootstown, Ohio, United States; j Department of Criminal Justice, Wayne State University, Detroit, Michigan, United States; k Department of Psychiatry and Behavioral Neurosciences, Wayne State University, Detroit, Michigan, United States; l Department of Community Health and Epidemiology, Dalhousie University, Halifax, Nova Scotia, Canada; m Department of Anesthesiology, Michigan Medicine, Ann Arbor, Michigan, United States; n Veterans Affairs Center for Clinical Management Research, Ann Arbor, Michigan, United States; o College for Health, Community and Policy, University of Texas-San Antonio, San Antonio, Texas, United States; p Department of Emergency Medicine, Yale School of Medicine, New Haven, Connecticut, United States; q Department of Emergency Medicine, Rutgers New Jersey Medical School, Newark, New Jersey, United States; r Department of Psychiatry and Behavioral Sciences, Johns Hopkins School of Medicine, Baltimore, Maryland, United States; s Department of Behavioral Medicine and Psychiatry, West Virginia University School of Medicine, Morgantown, West Virginia, United States; t Department of Biostatistics, Mailman School of Public Health, Columbia University, New York, New York, United States; u School of Nursing, Columbia University, New York, New York, United States

**Keywords:** Suicide, Injury, Mortality, Substance misuse, Drugs, Prevention, Poisoning, Mental disorders, Mental health

## Abstract

**Background:**

Suicides by any method, plus ‘nonsuicide’ fatalities from drug self-intoxication (estimated from selected forensically undetermined and ‘accidental’ deaths), together represent self-injury mortality (SIM)—fatalities due to mental disorders or distress. SIM is especially important to examine given frequent undercounting of suicides amongst drug overdose deaths. We report suicide and SIM trends in the United States of America (US) during 1999–2018, portray interstate rate trends, and examine spatiotemporal (spacetime) diffusion or spread of the drug self-intoxication component of SIM, with attention to potential for differential suicide misclassification.

**Methods:**

For this state-based, cross-sectional, panel time series, we used de-identified manner and underlying cause-of-death data for the 50 states and District of Columbia (DC) from CDC's *Wide-ranging Online Data for Epidemiologic Research*. Procedures comprised joinpoint regression to describe national trends; Spearman's rank-order correlation coefficient to assess interstate SIM and suicide rate congruence; and spacetime hierarchical modelling of the ‘nonsuicide’ SIM component.

**Findings:**

The national annual average percentage change over the observation period in the SIM rate was 4.3% (95% CI: 3.3%, 5.4%; *p*<0.001) versus 1.8% (95% CI: 1.6%, 2.0%; *p*<0.001) for the suicide rate. By 2017/2018, all states except Nebraska (19.9) posted a SIM rate of at least 21.0 deaths per 100,000 population—the floor of the rate range for the top 5 ranking states in 1999/2000. The rank-order correlation coefficient for SIM and suicide rates was 0.82 (*p*<0.001) in 1999/2000 versus 0.34 (*p* = 0.02) by 2017/2018. Seven states in the West posted *a* ≥ 5.0% reduction in their standardised mortality ratios of ‘nonsuicide’ drug fatalities, relative to the national ratio, and 6 states from the other 3 major regions *a* >6.0% increase (*p*<0.05).

**Interpretation:**

Depiction of rising SIM trends across states and major regions unmasks a burgeoning national mental health crisis. Geographic variation is plausibly a partial product of local heterogeneity in toxic drug availability and the quality of medicolegal death investigations. Like COVID-19, the nation will only be able to prevent SIM by responding with collective, comprehensive, systemic approaches. Injury surveillance and prevention, mental health, and societal well-being are poorly served by the continuing segregation of substance use disorders from other mental disorders in clinical medicine and public health practice.

**Funding:**

This study was partially funded by the National Centre for Injury Prevention and Control, US Centers for Disease Control and Prevention (R49CE002093) and the US National Institute on Drug Abuse (1UM1DA049412–01; 1R21DA046521-01A1).

**Research in context**

**Evidence before this study**

Multilevel, multivariable analysis of microdata from the National Violent Death Reporting System database indicated that suicides by drug self-intoxication are more difficult to detect for medical examiners and coroners in the United States of America (US) than suicides using behaviourally and forensically overt methods, such as shooting and hanging. Measuring self-injury mortality (SIM)—suicides plus estimated ‘nonsuicide’ drug self-intoxication deaths—circumvents such misclassification and more accurately accounts for fatal self-injuries. One previous study had respectively compared SIM and suicide in the US, by sex and race/ethnicity; another ranked SIM amongst the leading causes of death.

**Added value of this study**

Counting suicides alone as the measure of fatal self-injury in the US emphasizes a western states’ suicide belt. Measuring SIM and utilizing spacetime data, we can discern a burgeoning national mental health crisis that encompasses all 4 major geographic regions (Northeast, Midwest, South and West).

**Implications of all the available evidence**

Reversing the rising national SIM trends in the US will require collective, comprehensive and systemic approaches, a challenge further exacerbated by the COVID-19 pandemic. The continuing segregation of substance use disorders from other mental disorders in both public health practice and clinical medicine poorly serves efforts to enhance injury surveillance and prevention, mental health promotion, and societal well-being.

Alt-text: Unlabelled box

## 1 Introduction

Although highly conflated in reality [1], [2], suicides and fatal drug overdoses in the United States (US) have been treated as distinct phenomena in the scientific literature, mass media coverage, and governmental funding priorities. When viewed through an ecological lens, many of these deaths arise from common adverse life circumstances and personal distress, and are the result of motivated behaviour, even as medical examiners and coroners (ME/CS), as well as family members and other survivors, seek to disentangle and define the intent of decedents’ final moments [3,4]. Together with other colleagues, we have advocated the use of ‘self-injury mortality’ (SIM) to mitigate the uncertainties of injury manner of death determinations, while underscoring the collective public health importance of intervening long before people come to the ‘edge of the ledge’ [5,6]. Case and Deaton encompass SIM within their ‘deaths of despair,’ and emphasize the tragic economic circumstances that often contribute to the contextual underpinnings of recent decreases in US life expectancy [7].

Conceptually, SIM seeks to address two inadequately considered public health issues. Suicides and fatal drug overdoses frequently arise in common populations, together with even more primary medical fatalities reflecting the same risk behaviour [8,9]. While many of the factors leading to different final causes of death remain ill-defined, reducing mortality from all causes will require mitigation of their shared antecedent risks. Furthermore, once someone has died, SIM as a metric accommodates the fact that medicolegal assignment of most drug self-intoxication fatalities, without a readily definable indication of suicidal intent, as ‘accident’ mischaracterises the actions and circumstances immediately leading to many of these deaths [10]. Fatalities following motivated, repetitive use of potentially lethal agents are highly foreseeable; the probability of death had been fundamentally altered [11]—especially amongst those with opioid use disorder, where 58% entering treatment reported at least one prior non-fatal opioid overdose in one study [12] and in another 67% reported witnessing a drug overdose [13]. Without strong corroborative evidence indicating intent, such as an authenticated suicide note, documentation of a prior suicide attempt, or severe psychiatric comorbidity, suicides using drugs appear much more difficult for ME/Cs to determine than those by more forensically and behaviourally overt methods, most notably shooting and hanging [14,15,16,17]. This evidence typically is absent or deficient. In addition to these concerns, separating suicide and overdose fatalities into buckets or silos fails to adequately depict the extent of the epidemic of self-inflicted deaths in the US related to mental disorders and distress. Estimated self-injury accounts for more premature mortality nationally than do diabetes, influenza and pneumonia, or kidney disease [5].

In this observational study, we first mapped and graphed the magnitude and growth of suicide versus SIM rates across states during the period 1999–2018. States are responsible for compiling mortality and other vital statistics, which they then forward to the US National Centre for Health Statistics for purposes of generating national and comparative state mortality profiles and reports and informing research, prevention, treatment and evaluation. Secondly, we conducted a spatiotemporal analysis of the diffusion or spread of the motivated, self-injurious, ‘nonsuicide’ drug component of SIM, relative to the nation as a whole, which emphasised regional clustering as well as individual states. Preliminary to this analysis, we plotted changes by state in the proportion of SIM attributable to the ‘nonsuicide’ component. Owing to the formidable medicolegal challenges to ascertaining drug suicide cases, gross interstate and regional variation in the magnitude and changes in the diffusion and the proportion of ‘nonsuicide’ SIM has implications for differential suicide detection. Research on SIM and suicide takes on added urgency during the COVID-19 pandemic and concomitant economic recession [18], given increasing drug overdose rates [19].

## 2 Methods

### 2.1 Data sources and SIM operationalisation

In this state-based, cross-sectional, panel time series, we used deidentified manner and underlying cause-of-death data and associated population data for 1999–2018 for all 50 US states and the District of Columbia (DC) from the Centers for Disease Control and Prevention's (CDC's) *Wide-ranging ONline Data for Epidemiologic Research* (WONDER) [20]. Nosologists precoded certified deaths according to the *ICD-10* [21]. SIM is a composite of all suicides (ICD-10 UO3, X60-X84, Y 87.0) by any method, irrespective of decedent age, and 80% of accidental (‘unintentional’ under CDC nomenclature) opioid and other drug intoxication deaths (X40-X45) and 90% of corresponding deaths of undetermined intent (Y10–15) amongst persons ages 15 years and older. We included alcohol poisoning deaths when operationalising SIM, a change from prior studies [5,6]. Our fraction of undetermined deaths for inclusion in SIM was higher than that of ‘accidents’ for two reasons. Unlike accident, an undetermined assignment by ME/Cs allows for the possibility that the true injury manner of death is suicide, accident or homicide. Since these officials determined homicides comprised 0.2% of drug fatalities during 2009–2018, versus 10% for suicides and 84% for accidents [20], we assumed suicide and accident were the main competing options within the undetermined category (6%).

We assuredly incorporate undetected, misclassified suicides (false negatives) within SIM through our inclusion of the selected ‘accident’ and undetermined drug deaths. Our assignment of SIM is predicated on the presence of repetitive self-harm behaviours, which are commonly associated with substance use disorders, even as the great preponderance of drug deaths do not meet the stringent criterion of establishing decedent intention to die [10]. In contrast, a non-SIM drug fatality could involve an unanticipated fatal interaction between a prescribed opioid and other prescribed medications. The relative rarity of repetitive, purposive, drug-related behaviour amongst pre- and younger teens [22] motivated our 15-year old age cutoff for the ‘nonsuicide’ drug component in the SIM estimates. Representing the actual burden of fatal self-injury to the nation and states, we used crude rather than age-adjusted suicide, ‘nonsuicide’ drug self-intoxication death and SIM rates that applied a hypothetical population, such as the *Year 2000 Standard*.

### 2.2 Joinpoint regression and rate trends

In describing national suicide and SIM crude rate trends, we employed the Joinpoint Regression Program software, version 4.6.0.0 [23], to identify inflection points where respective trends changed significantly during the observation period. Joinpoint software fitted weighted least-squares regression models to the rates on the log-transform scale. Selection of joinpoints (trend inflections) was based on a permutation test at an overall significance level of 0.05. Elaborated upon in Appendix 1 in an online supplement, along with the test statistic and additional results, this methodology provided the annual percentage change in rates between trend-change points, and the average annual percentage change during the total 20-year observation period, plus associated 95% confidence intervals (CIs).

### 2.3 Interstate rate stabilisation and congruence assessment

To stabilise the interstate suicide, estimated ‘nonsuicide’ SIM and total SIM data, we computed two-year annual averaged rates, proportions and counts for mapping, graphing and statistical modelling purposes. Our state-based trend data integrated intermediate observation points detected by the national joinpoint regression analysis. We calculated Spearman's rank-order correlation coefficients to examine congruence between corresponding ranked suicide and SIM rates.

### 2.4 Spacetime hierarchical Bayesian modelling of ‘nonsuicide’ drug self-intoxication deaths

Mapping suicide and SIM rates by state enables us to observe spatial clusters. However, since the 48 states and DC in the contiguous US variously share boundaries with other states (and with Canada and Mexico), their rates do not reflect statistical independence. Some have multiple borders; for example, Tennessee and Missouri each have eight neighbouring states. They consequently generate more shared information for measuring random variation in ‘nonsuicide’ drug fatalities. In modelling and mapping true spacetime trends of these overdose deaths, we conducted Bayesian Hierarchical Modelling (BHM) employing a log-normal Poisson distribution using the R–INLA package [24]. Standardised mortality ratios (SMRs) were estimated for each state by dividing the state-level ‘nonsuicide’ drug fatality rate per 100,000 by the national rate. Smoothed through a quadratic kernel estimation of the Bayes SMRs, we also generated a corresponding ‘heatmap’ of spacetime changes. For readability and economy, details on the derivation of the SMRs are reported in Appendix 2 in the online supplement, together with computer code to enable replication and adaptation. Associated measures, test statistics, quantiles and p-values are tabulated in that appendix, and spacetime data files are also included in the supplementary materials.

This study subscribed to *Strengthening the Reporting of OBservational studies in Epidemiology (STROBE)* guidelines [25]. As a secondary analysis of an aggregated, state-level, publicly accessible mortality dataset, it was exempted from an ethical evaluation by the Institutional Review Board of West Virginia University.

### 2.5 Role of the funding source

The funders had no role in the design and conduct of the study; collection, management, analysis, and interpretation of the data; preparation, review, or approval of the manuscript; and decision to submit the manuscript for publication.

## 3 Results

### 3.1 National suicide and SIM rates, 1999–2018

**Fig. 1:**
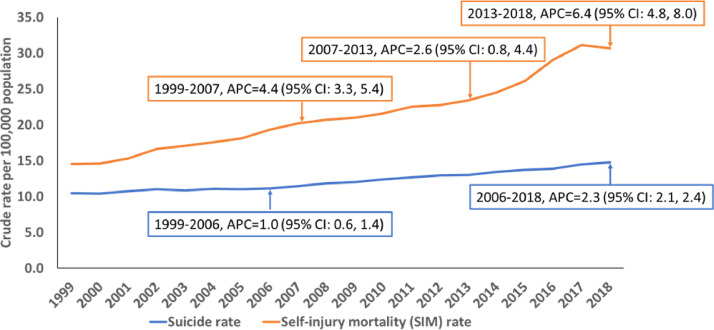
Trends and Significant Changes in Crude Suicide and Self-injury Mortality (SIM) Rates per 100,000 population, United States, 1999–2018. Note: APC=annual percentage change based on joinpoint regression.

The national crude suicide rate trended upwards from 1999 through 2018, where the rate at 14.8 per 100,000 population in 2018 was 1.41 times higher than the 1999 rate (Fig. 1). Its average annual percentage change was 1.8 (95% CI: 1.6%, 2.0%; *p*<0.001). Joinpoint regression analysis revealed one significant change in the upwards trajectory during the observation period. The annual suicide rate increased by 1.0% (95% CI: 0.6%, 1.4%; *p*<0.001) between 1999 and 2006, and more than doubled to 2.3% (95% CI: 2.1%, 2.4%; *p*<0.001) between 2006 and 2018.

At 35.1 deaths per 100,000, the national SIM rate in 2017 was 2.3 times higher than in 1999; the average annual percentage change between 1999 and 2018 was 4.3 (95% CI: 3.3%, 5.4%; *p*<0.001). There was a marginal rate decline of 1.6% between 2017 and 2018. The regression analysis also identified two significant inflection points in the rising trend of the SIM rate. This rate rose by 4.4% (95% CI: 3.3%, 5.4%; *p*<0.001) annually between 1999 and 2007, slowing to 2.6% (95% CI: 0.8%, 4.4%; *p*<0.001) between 2007 and 2013, and then increasing 2.5 times to 6.4% (95% CI: 4.8%, 8.0%; *p*<0.001) annually between 2013 and 2018.

### 3.2 Geographic visualisation of suicide and SIM rate changes by state, region and period

**Fig. 2:**
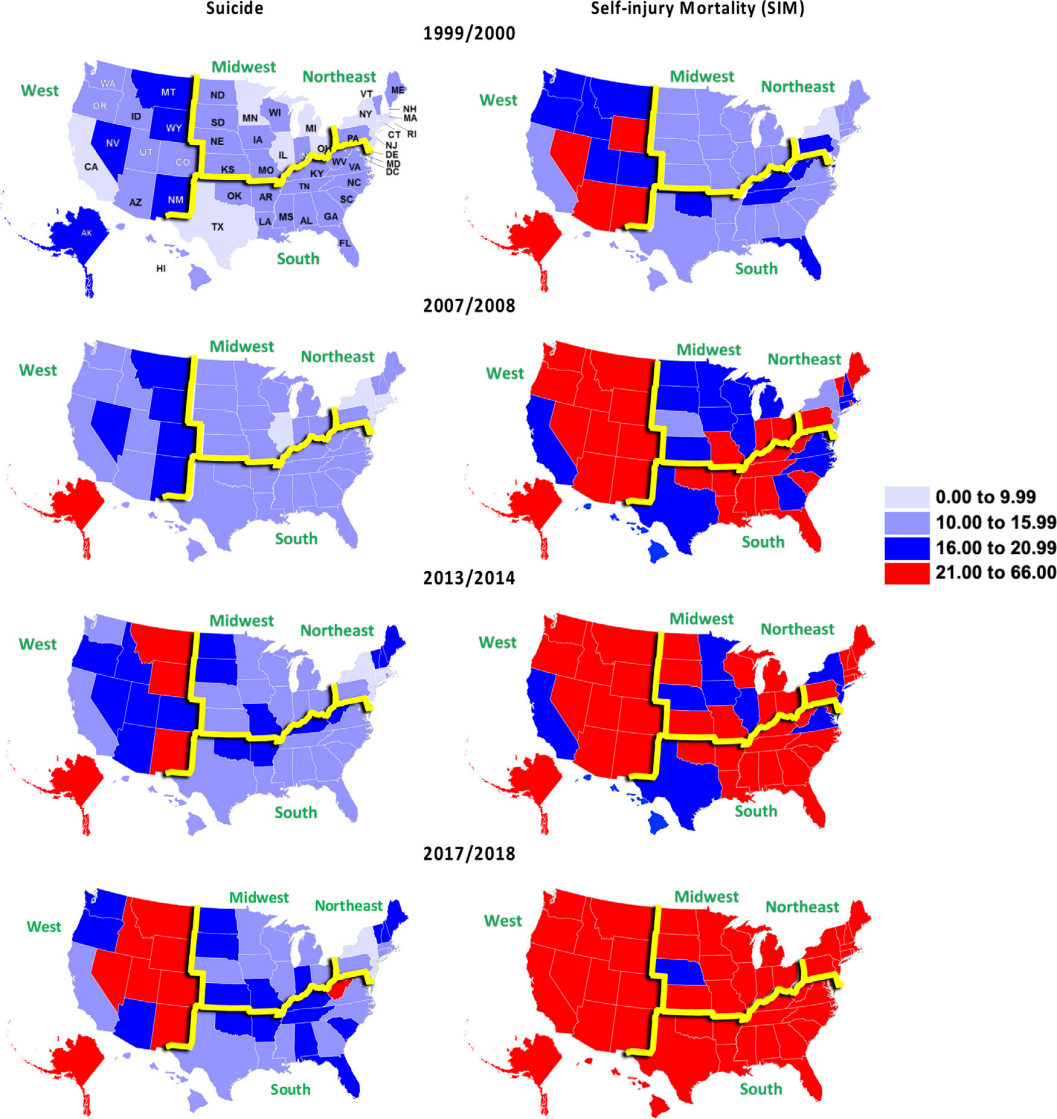
Mapped Annual-averaged Crude Suicide and Self-injury Mortality (SIM) Rates per 100,000 Population by State and Period, United States.

Maps portray suicide and SIM rates per 100,000 population for states, plus DC, within the 4 major regions of the US across space and time (Fig. 2). The 5 states with the highest estimated SIM rates in 1999/2000—Nevada, New Mexico, Alaska, Arizona and Wyoming, all located in the West—serve as referents for suicide as well as SIM changes, in depicting the intensification of fatal self-injury across the entire observation period. Their range extended from 21.0 deaths per 100,000 population to 28.6. By 2017/18, relatively high SIM rates enveloped the nation. Only Nebraska reflected a lower rate than that of the fifth-ranked state, Wyoming, in 1999/2000—19.9 versus 21.0 per 100,000. When fatal self-injury was represented by suicide alone, Alaska was the first state at any of our observation points whose suicide rate entered the SIM rate range for the 5 referents in 1999/2000. That occurred in 2007/08. By 2017/18, 8 western states—Alaska, Montana, New Mexico, Wyoming, Idaho, Colorado, Nevada and Utah—and West Virginia occupied this range. Except for Alaska, suicide rates in the western coastal states tended to be lower than in the remainder of the region.

### 3.3 Graphing interstate suicide and SIM rates

**Fig. 3:**
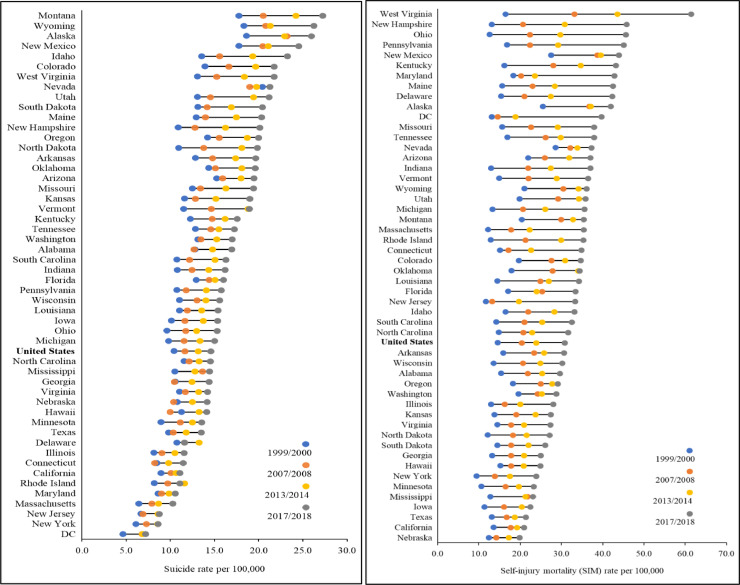
Graphed Annual-averaged Crude Suicide and Self-injury Mortality (SIM) Rates by State and Period, United States.

Lollipop graphs complement the maps in displaying the interstate suicide and SIM rates over time (Fig. 3). New Mexico alone ranked amongst the 5 states with both the highest suicide and SIM rates in 1999/2000, 2007/08, 2013/14 and 2017/18. There was no parallel in the corresponding tier of states with the lowest rates. Four western states—Alaska, Montana, New Mexico and Wyoming—ranked amongst the 5 states/territory with the highest suicide rates at all 4 observation points. Three northeastern states—New Jersey, New York and Massachusetts, together with DC—remained in the lowest rate echelon throughout. Registering the highest suicide rate in 2017/18, Montana ranked twenty-first on SIM. Whereas DC posted the lowest suicide rate, it ranked eleventh on SIM. The state with the highest SIM rate, West Virginia, had the seventh highest suicide rate. The state with the lowest SIM rate, Nebraska, ranked thirty-eighth on suicide. Signifying relatively high congruence, the values of Spearman's rank-order correlation coefficients for ranked suicide and SIM rates across the states and DC in 1999/2000 and 2007/08, respectively, were 0.82 (*p*<0.001) and 0.86 (*p*<0.001). The coefficient declined marginally to 0.75 (*p*<0.001) in 2013/14, and then precipitously to 0.34 (*p* = 0.02) in 2017/18.

### 3.4 Distribution and diffusion of ‘nonsuicide’ drug self-intoxication deaths

**Fig. 4:**
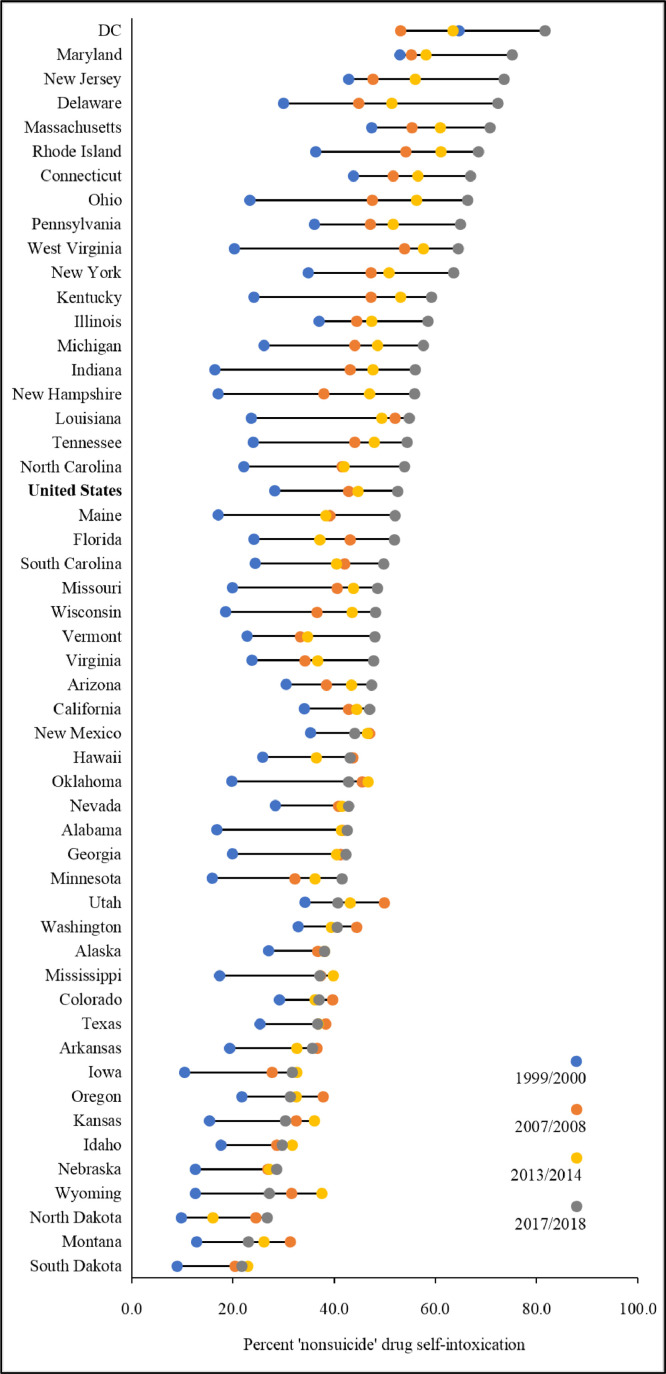
Annual-averaged Percentage of ‘Nonsuicide’ Drug Self-intoxication Deaths of Self-injury Mortality (SIM) by State and Period, United States.

Fig. 4 displays the distribution of the proportion of SIM accounted for by estimated ‘nonsuicide’ drug self-intoxication fatalities across states and the 4 observation points. For example, respective ranges extended from 9.0% to 64.7% in 1999/2000 and from 21.8% to 81.8% in 2017/18, with South Dakota and DC positioned at the respective lower and upper limits in both instances. DC, Maryland and Massachusetts ranked near the ceiling of the range at all 4 observation points on the ‘nonsuicide’ drug self-intoxication death/SIM metric, and South Dakota, North Dakota, and Nebraska near the floor.

**Fig. 5:**
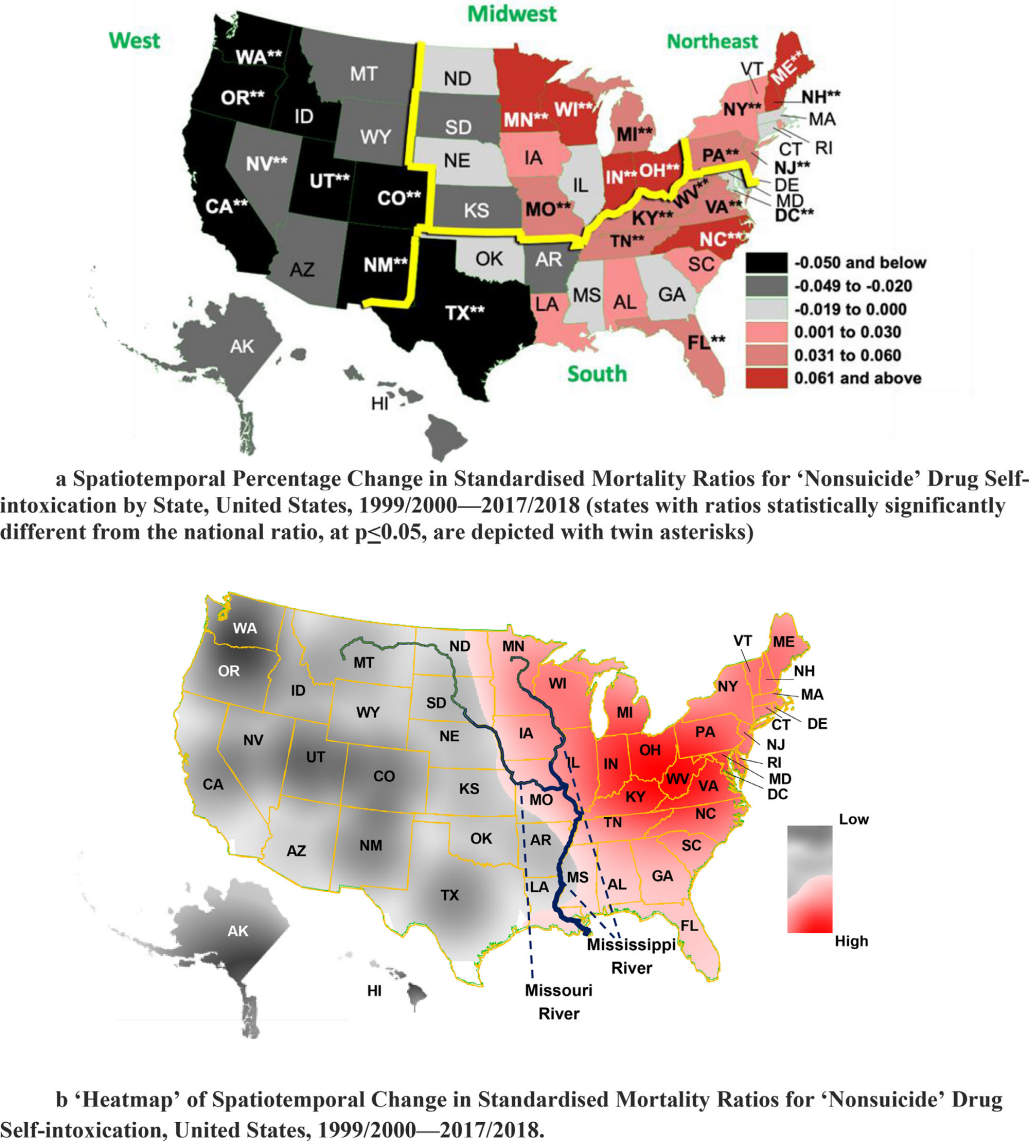
a Spatiotemporal Percentage Change in Standardised Mortality Ratios for ‘Nonsuicide’ Drug Self-intoxication by State, United States, 1999/2000—2017/2018 (states with ratios statistically significantly different from the national ratio, at *p* ≤ 0.05, are depicted with twin asterisks). Fig. 5b ‘Heatmap’ of Spatiotemporal Change in Standardised Mortality Ratios for ‘Nonsuicide’ Drug Self-intoxication, United States, 1999/2000—2017/2018.

Turning to our comparative measures of diffusion, spacetime expansion in standardised ‘nonsuicide’ drug mortality ratios (SMRs) was below the national average between 1999/2000 and 2017/18 for western states, higher in most midwestern and northeastern states, and close to the average in an excess of southern states (Fig. 5a). State SMRs were adjusted for this spacetime bias to reflect their true values. Texas, New Mexico, Colorado, Utah, Oregon, California and Washington manifested a reduction of at least 5% in respective SMRs relative to the ratio for the nation over the four observation points (*p*<0.05). Minnesota, Wisconsin, Indiana, Ohio, North Carolina and Maine, by contrast, showed a corresponding 6.1% or higher relative increase (*p*<0.05). The ‘heatmap’ exposes a national divide along the Missouri and lower Mississippi rivers. There was contraction of standardised ‘nonsuicide’ drug self-intoxication mortality ratios in western states and expansion in eastern states relative to the nation (Fig. 5b).

## 4 Discussion

Although the data also spotlight a previously documented suicide belt in the West [26], our substitution of SIM for suicide—as the representative of fatal self-injury and imminent personal and societal distress—highlights a mental health crisis that is national rather than regional in geographic scope. Contrasting with singular western representation in 1999/2000, the five states with the highest SIM rates in 2017/18 comprised two from the Northeast (New Hampshire and Pennsylvania) and one each from the South (West Virginia), Midwest (Ohio) and West (New Mexico). Our view that drug intoxication deaths are a constituent of the mental health domain conforms to the inclusion and classification of substance use disorders in the *Diagnostic and Statistical Manual of Mental Disorders* (DSM-5) [27] and earlier versions.

While the suicide rate in the US increased by 41% between 1999 and 2018, the SIM rate grew much more rapidly at 128%. We suggest this expanding SIM/suicide rate gap over the observation period partially reflects increasing difficulties for ME/Cs to establish suicide as a manner of death under the strain of the opioid epidemic [28]. Warranting in-depth analytic research for confirmation or refutation, states appearing relatively strong in suicide detection, based on the 2017/18 mortality data, include North and South Dakota, Montana, Wyoming and Nebraska, as compared to those with apparently weak detection, such as DC, Maryland, New Jersey, Delaware, and Massachusetts. Besides being less impacted by the opioid epidemic [29], plausibly facilitating better case ascertainment of suicides during the observation period by ME/Cs in the western states, *vis-à-vis* those on the north-eastern seaboard, is the greater prominence of firearm use [30]—as a forensically and behaviourally overt method—and consistent with their profoundly higher gun ownership rates [31]. We note, however, that data released by CDC on December 17, 2020 point to a substantial rise in synthetic opioid driven overdose fatalities in the West [19].

State rank-order comparisons of SIM and suicide rates, aligned with the inflections identified in the national SIM trend analysis, showed the most pronounced change in the SIM/suicide rate gap occurred between 2013/14 and 2017/18. This change coincided with a sharp rise in deaths attributable to illicit fentanyl and other synthetic opioids—highly lethal compounds that have contributed to the rapid acceleration in death rates [32], and likely further exacerbating investigative challenges faced by ME/Cs [33]. Behaviourally such deaths qualify as SIM, even with no medicolegal corroboration of suicide. Another inflection point in SIM rates coincided with the onset of the 2007/08 ‘Great Recession,’ an event previously associated with elevated suicide rates [34].

Based on the intersection of the continuing epidemic of SIM with the COVID-19 pandemic, we are concerned the release and analysis of 2020 underlying cause of death data will disclose new inflection points in the rates of both SIM and suicide—informed by a three-fold increase in self-reported serious psychological distress between 2018 and April 2020 [35]. A recent commentary characterised the COVID-19 pandemic as creating a ‘perfect storm’ for suicide [36]. An early indication of rising SIM [19] reinforces our concern that the current escalations in personal and societal distress will be critical drivers of such preventable deaths. Improved modelling and robust prevention and early intervention efforts are needed urgently. Our data argue strongly for fundamentally reassessing the problematic conceptual separations of substance use disorder-associated deaths from other mental health disorder-associated deaths—especially pertinent to surveillance and prevention initiatives, to systems for providing health care, and to research funding. These conditions have been placed in artificial silos that segregate convergent and co-occurring disorders. Some separations associated with SIM are logical; for example, injection drug use is associated with infectious diseases (e.g., HIV, hepatitis C, endocarditis) that uniquely elevate all-mortality risk [37,38]. By contrast, episodic desire to die is commonly present amongst both individuals with substance use disorders and individuals with a broad array of mental health disorders, including drug users who have survived a near-fatal overdose [39,40].

The SIM epidemic—including suicides and drug poisoning fatalities—will not be reversed merely by interventions nested within the healthcare system. SIM and suicide interventions must also address well-described upstream social determinants [3,4], and involve major and integrated structural and public policy changes throughout the economic, political, educational, policing and criminal justice, environmental protection, and healthcare systems.

This study has several limitations. We could not assess interstate heterogeneity in the quality of medicolegal death investigations [14], and our ecological study obscured any within-state variations (e.g., urban versus rural). Yet another limitation, we did not incorporate all potential self-injury deaths, such as some ‘accidental’ drowning, cutting, and motor vehicular deaths that could be linked aetiologically to misuse of alcohol and other psychoactive substances [5]. Currently inestimable, we predict they would be relatively rare compared to deaths where drug overdose was the underlying cause. The diffusion of ‘nonsuicide’ drug fatalities across space and time demands in-depth investigation, with consideration of such factors as migration and the psychological influence of social and mass media, since understanding would facilitate framing, designing, and targeting interventions.

More fundamentally, SIM remains an indirect measure. It can be critiqued as an estimation, albeit based on likely conservative estimates of ‘nonsuicide’ drug self-intoxication deaths. A further critique would support the continued separate tracking of drug-related mortality and suicide. However, this practice fails to underscore the urgent public health response needed to address the rising tide of deaths caused by self-directed injurious behaviour—with emphasis on the impact of fatal actions rather than medicolegal discernment of the final intentions of decedents. Indeed, SIM more accurately captures the impacts of personal and societal distress than suicide or overdose mortality measures alone and, in so doing, reflects that the medicolegal interpretation of most fatal nonsuicidal drug overdoses as ‘accidents’ is a mischaracterisation for prevention, treatment and evaluation purposes, even while appropriate under extant protocols that guide medical examiners and coroners in assigning manner of death.

Towards direct accounting of SIM and validation or refinement of our estimates of ‘nonsuicide’ drug self-intoxication mortality, a case has been presented for adding a checkbox to the death certificate for recording self-injury status, regardless of whether manner of death was suicide, accident or undetermined [41]. As a first step, routine collection and reporting by ME/Cs of mortality data that indicate opioid and other psychoactive drug misuse, would form a firm foundation for distinguishing self-injury. Enumerated in a 2020 position paper from the National Association of Medical Examiners and the American College of Toxicology, death scene findings suggesting opioid misuse, which we would characterize as examples of SIM, include “Evidence of intravenous drug use (needles, cooker spoons, tourniquet, crushed tablets, packets of powder or crystals, other drug paraphernalia); evidence of insufflation (chopped pills or residue, chopped lines, cuts on coffee table glass, cut straws or pen tubes, rolled bills, etc.); overlapping prescriptions for the same type of prescribed controlled substances, prescriptions for controlled substances from multiple pharmacies or multiple prescribers; prescriptions in other people's names; pills not stored in prescription vials or mixed in vials; injection sites not due to resuscitation attempts; altered transdermal patches; many transdermal patches on the body or transdermal patches in unusual locations, e.g., mouth, stomach, vagina, or rectum; application of heat to increase the rate of transfer of drug from transdermal patch to decedent; (and the) presence of naloxone [42].”

Depiction of rising SIM trends across states and major regions unmasks a burgeoning national mental health crisis. Geographic variation is plausibly a partial product of heterogeneity in local forces, such as toxic drug availability and the quality of medicolegal death investigations. Like COVID-19, the nation will only be able to prevent SIM by responding with collective, comprehensive, systemic approaches. Injury surveillance and prevention, mental health, and societal well-being are poorly served by the continuing segregation of substance use disorders from other mental disorders in clinical medicine and public health practice.

## Funding

This study was partially funded by the National Centre for Injury Prevention and Control, US Centers for Disease Control and Prevention (R49CE002093) and the US National Institute on Drug Abuse (1UM1DA049412-01; 1R21DA046521-01A1).

## Data sharing

The primary data source for this study is the US Centers for Disease Control and Prevention's underlying cause-of-death data accessible through the *Wide-ranging ONline Data for Epidemiologic Research* (WONDER) database: https://wonder.cdc.gov/ucd-icd10.html. Details on the joinpoint regression and spacetime analyses are accessible in the online supplementary materials.

## Author contributions

IRHR conceived and coordinated the study and acquired the data. IRHR and AB designed the study. IRHR, AB, EDC and HSC searched the literature. BA, AB and VOL prepared the figures. IRHR, EDC, AB, BA, TM, HSC, KBN, RKM and HJ wrote the first draft report. IRHR, EDC, AB, BA, TM, VOL, GLL, SS, BH and HJ contributed to the analysis. IRHR, EDC, AB, BA, TM, HSC, VOL, KBN, GLL, SS, BH, RKM, FMMW, SFG, ASBB, JSC, GD, LSN, PSN, JHB and HJ contributed to the interpretation. IRHR, EDC, TM, HSC, KBN, GLL, BH, RKM, FMMW, SFG, ASBB, JSC, LSN and JHB edited the report. IRHR had the final responsibility for the decision to submit for publication. All authors critically reviewed the report and approved the final version. IRHR and AB verified the underlying data.

## Declaration of Competing Interest

BA and TM report litigation support contract research funding from Plaintiff Attorneys in Government Opioid Litigation, outside the submitted work; JHB was a scientific advisor for Celero, Inc., outside the submitted work.

## Appendix Supplementary materials

Image, application 1

Image, application 2

Image, application 3
