# Supplementary material for: Fatal self-injury in the United States, 1999–2018: Unmasking a national mental health crisis
Source: eClinicalMedicine. 2021 Feb 8;32:100741. doi: 10.1016/j.eclinm.2021.100741 (PMC7910714; doi:10.1016/j.eclinm.2021.100741)
Supplement: Supplementary file 1 [file mmc1.docx]

**Appendices 1 & 2**

**[Fatal Self-Injury in the United States, 1999-2018: Unmasking a National Mental Health Crisis]**

**Appendix 1: Joinpoint Regression**

We employed the Joinpoint Regression Program, version 4.6.0.0,^1^ to identify inflection points during the observation period where respective suicide and self-injury mortality (SIM) rates changed significantly. Joinpoint software fitted weighted least-squares regression models to the rates on the log-transform scale. Selection of joinpoints (trend inflections) was based on the permutation test at an overall significance level of 0.05. Improving the accuracy of the trend data, the inflection points demand representation by continuous linear segments instead of a single slope.^2^ A general regression model can be expanded to represent the joint points. Typically, a simple model is specified as follows: $Y_{t}= \alpha+ \beta*Y_{t-1}$, where $f\left( x \right)$ and $X$ are related by factor $\beta$. However, in joinpoint regression, we can expand $\beta$ to incorporate several values. For example, 2 or more different $\alpha^{'}s$ and $\beta's$ would result when $X$ changes before and after say, time ‘t’:^3^

$\left\{ \begin{aligned} \alpha_{1}+ \beta_{1}*Y_{t-1} &x<t_{1} \\ \alpha_{2}+ \beta_{2}*Y_{t-2} &x<t_{2} \\ \alpha_{3}+ \beta_{3}*Y_{t-3} &x<t_{3} \\ \begin{aligned} \ldots\ldots\ldots\ldots\ldots\ldots\ldots..\ldots\\ \alpha_{k}+ \beta_{k}*Y_{t-k} &x<t_{k} \end{aligned} \end{aligned} \right\}$…………………………………………………………………..(1)

The results of the respective suicide and SIM trend analyses are presented in Tables 1 and 2 following the listing of the references.

**References**

1. National Cancer Institute. *Joinpoint Regression Program.* Surveillance Research Program, Statistical Methodology and Applications Branch; 2018.

2. Kim H-J, Fay MP, Feuer EJ, Midthune DN. Permutation tests for joinpoint regression with applications to cancer rates. *Statistics in Medicine*. 2000;19(3):335-351. doi:10.1002/(SICI)1097-0258(20000215)19:3<335::AID-SIM336>3.0.CO;2-Z

3. Martínez-Beneito MA, Botella-Rocamora P. *Disease Mapping: From Foundations to Multidimensional Modeling*. CRC Press; 2019.

4. Blangiardo M, Cameletti M. *Spatial and Spatio-Temporal Bayesian Models with R-INLA.* John Wiley & Sons; 2015.

5. Van den Broeck J, Brestoff J, Kaulfuss C. Statistical estimation. In: *Epidemiology: Principles and Practical Guidelines.* Springer Netherlands; 2013:417-438.

6. Knorr-Held L, Besag J. Modeling risk from a disease in time and space. *Statistics in Medicine*. 1998;17(18):2045-2060.

7. Bakka H, Rue H, Fuglstad G-A, et al. Spatial modelling with R-INLA: A review. Published online February 18, 2018. http://arxiv.org/pdf/1802.06350

8. Knorr-Held L. Bayesian modeling of inseparable space-time variation in disease risk. *Statistics in Medicine*. 2000;19(17/18):2555-2567.

**Table 1. Suicide Trend, United States 1999-2018, Joinpoint Results Output (Screenshot)**


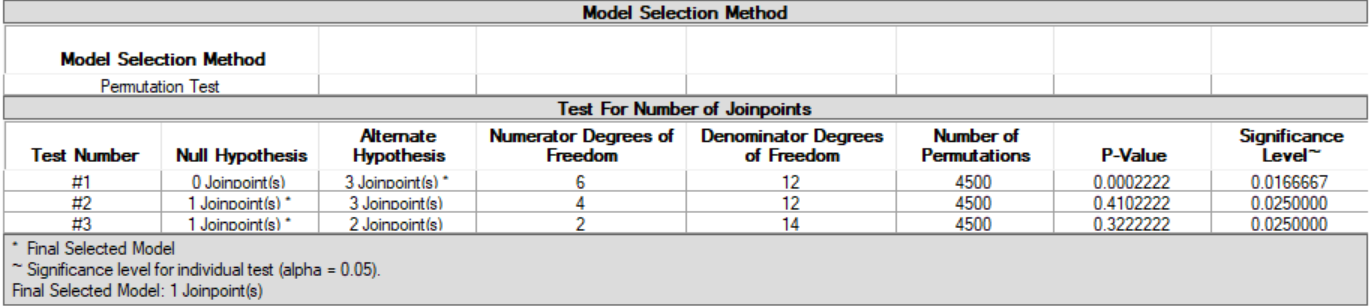


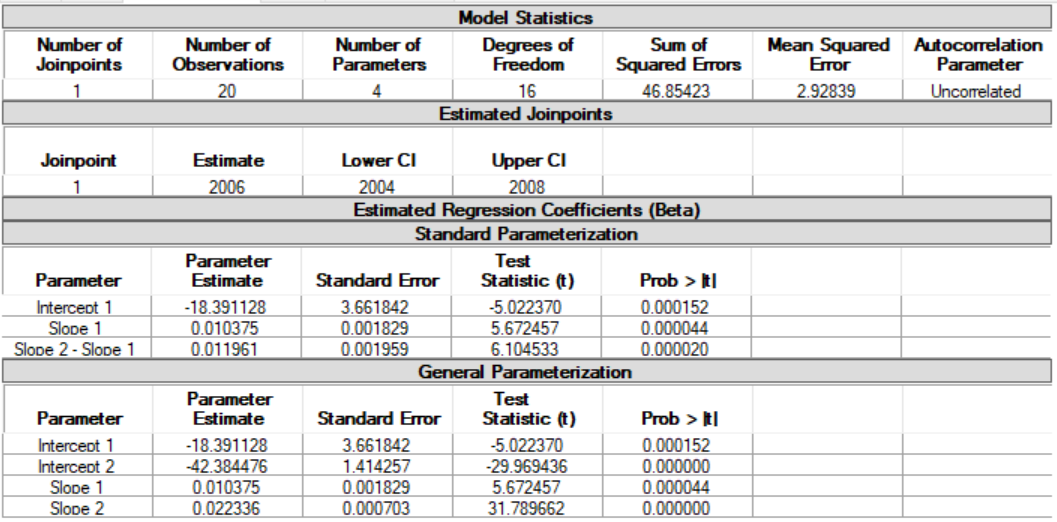


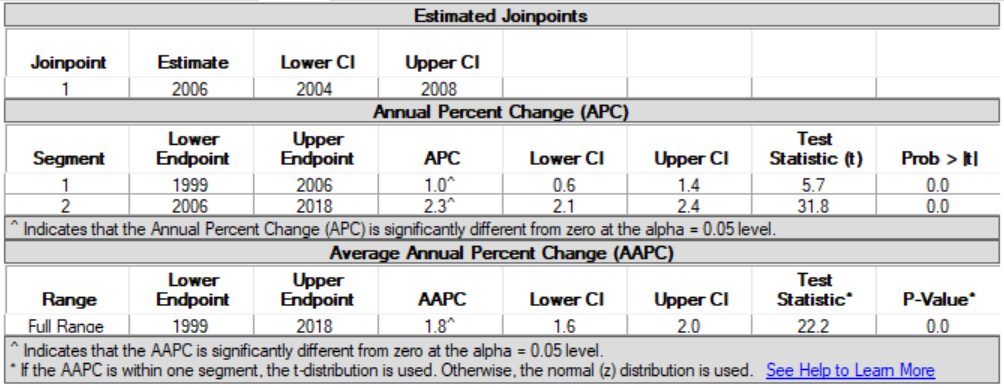


**Table 2. Self-injury Mortality (SIM) Trend, United States 1999-2018, Joinpoint Results Output (Screenshot)**


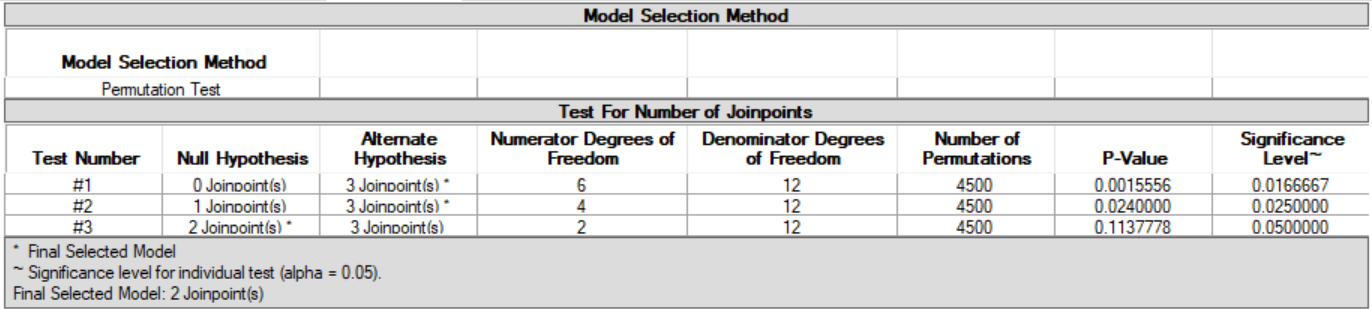


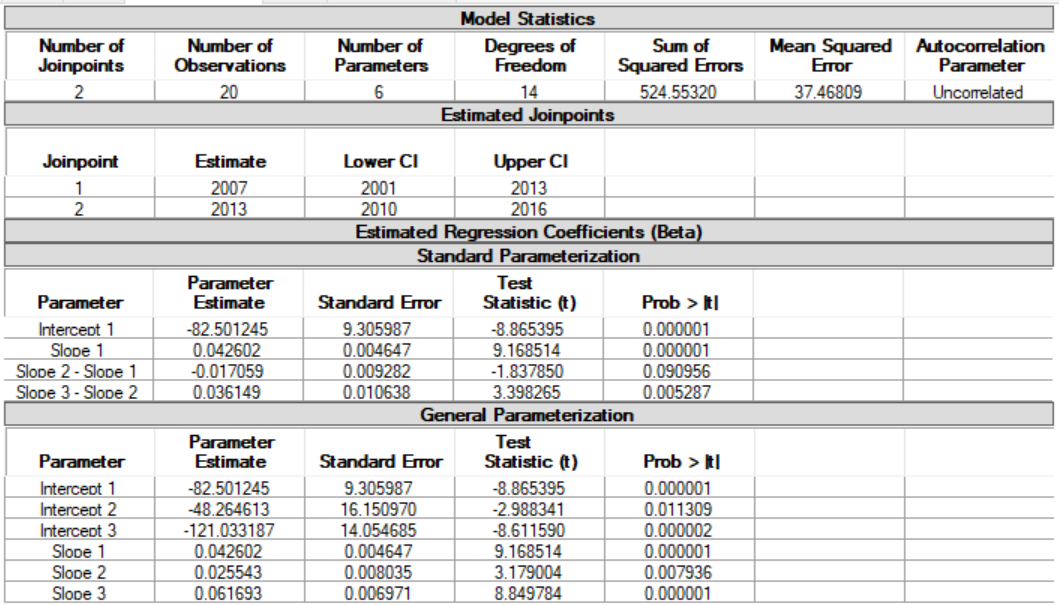


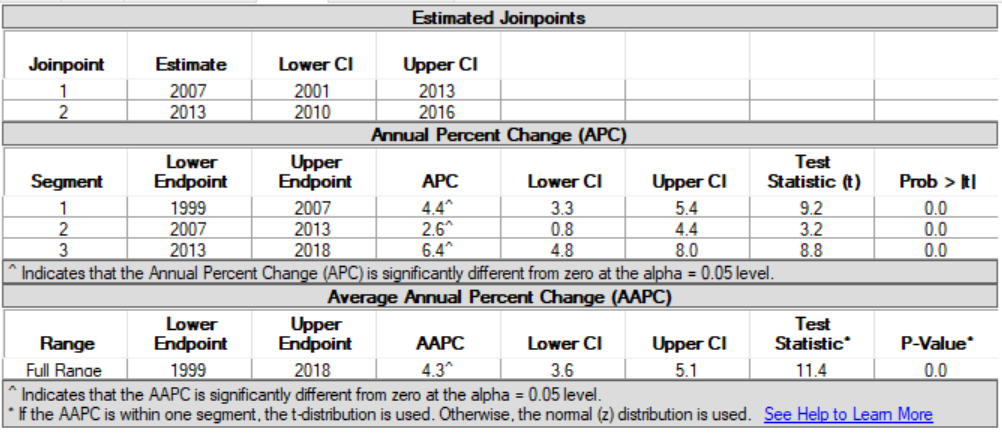


**Appendix 2: Spacetime Modeling and Code**

To model true space-time trends of ‘nonsuicide’ drug self-intoxication mortality, we conducted spatio-temporal Bayesian Hierarchical Modeling (BHM) applying a log-normal Poisson distribution using the R–INLA package.^4^ Standardized mortality ratios (SMRs) were estimated for each state by dividing the state-level ‘nonsuicide’ drug self-intoxication death rate by the rate per 100,000 for the United States of America (US).^5^ Specifications for state-level SMRs are shown below:

$\theta_{i}\left[ Standardized{{}^{'}{nonsuicide}}^{'}drug self-intoxication mortality ratio \right]=$

$\frac{Observed}{Expected}=\frac{Y_{i}}{E_{i}}$;….……..………………………………...(2a) where:

Y_i_= observed number of ‘nonsuicide’ drug self-intoxication deaths by state

E_i_= expected number of ‘nonsuicide’ drug self-intoxication deaths by state

*_i_* = 1, 2, 3, ….51 states (including DC as a state)

Calculating E_i_: We determined the at-risk population (N) to be all self-injury deaths (SIM or ‘Y’) for the US at each observation point, and then calculated the overall ‘nonsuicide’ drug self-intoxication mortality rate (‘R’). $R= \frac{\sum Y}{\sum N}$…………………………………………….…………………………………………….…....(2b)

The expected number (E) of ‘nonsuicide’ drug self-intoxication deaths per state(i) was derived by multiplying the rate by total SIM: $E_{i}= R*N_{i}$…………………………………………………….….(2c)

State-level SMRs were modeled directly using the BYM (Besag, York & Mollie) spatial Poisson regression, assuming a log-normal Poisson distribution for ‘nonsuicide’ drug self-intoxication deaths defined as: $y_{i}= Poisson \left( e_{i}*\theta_{i} \right)$………………………………………………….………….……..(2d)

$\theta_{i}= \frac{Y_{i}}{E_{i}}$……………………………….…………..………..(2e)

${log(\theta}_{i}or '{SMR}_{i}')= \alpha+u_{i}+v_{i}$…………………….…………………..(2f)

where $u_{i}$ was correlated spatial heterogeneity and $v_{i}$ was uncorrelated spatial heterogeneity. This BYM model employed an intrinsic conditional autoregressive structure (ICAR),^6^ based on a random walk primitive. It is a commonly used spatial model where administrative boundaries, like counties or states, are indexed discretely.^7^ Elements reflect variation not captured by $\alpha$ (the expected or mean SMR). By extending the BYM spatial model to temporal data (an interaction model was defined using Kronecker products), we got:

Q_spacetime_ = Q_time_$\otimes$Q_Space_……………………………………………..(2g)

Then, by stating that spacetime risks were inseparable,^8^ we obtained:^4^

${log(\theta}_{i}or 'SMR')= \alpha+u_{i}+v_{i}+\beta*t+\delta_{i}*t$……………………….…...(2h)

$\beta*t$ is the trend for the ‘nonsuicide’ drug self-intoxication deaths over the selected time points; $\delta_{i}*t$ provided the spacetime interaction bias or diffusion; $\delta_{i}$ captured only the variation that could not be explained by the main effects;^8^ $\delta_{i}<0$ represented a contraction of the ‘nonsuicide’ drug self-intoxication SMRs within and among adjacent states relative to total or national SIM; and $\delta_{i}>0$ represented a relative expansion of the ‘nonsuicide’ drug self-intoxication SMRs within and among adjacent states.

Equations 2a-h provide both space and space-time measures, and equation 2f the spatial variation measure for both structured and unstructured heterogeneity. The differential time effect (equation 2i) provided a measure of diffusion of deviation from the US as a whole. This effect was over and above the average or national SMR when there was no spacetime autocorrelation; i.e., the states were ‘islands’ and temporally uncorrelated (in this case just $e^{'\alpha'}$, the expected or mean SMR):

*differential time effect (diffusion)* =$e^{\delta_{i}*t}$ …………..……………………..(2i)

Smoothed through a quadratic kernel estimation, we also included a corresponding ‘heatmap’ in mapping spatio-temporal change in the SMRs.

**Significance of the spatial effects:**
To assess statistical significance, we looked at the 2.5% and 97.5% quantiles of diffusion for each state (fig 5a) ...  “If for some of these, both quantiles have the same sign, then the corresponding [random effects] are significantly different from zero."^4^ (Page 278)

**Computer Code**

**require**("rgdal")
**require**("spdep")
**require**("sp")
**require**("raster")
**require**("INLA")
**inla.setOption**(scale.model.default=FALSE)
**require**("splancs")
**require**("lattice")
**require**("abind")
**require**("maptools")

suicidedata <- **read.csv**("for_Rz4.csv")
statesshp <- **readShapePoly**("statesshp.shp")

*## since data and shapefile are not ordered correctly we have to reorder them*
*## First let's fix some errors*
*## Since there are issues with "D C" and "New Hamphsire" fix them...*
statesshp**@**data**$**STATENAME <- **gsub**("D C", "DC", **as.character**(statesshp**@**data**$**STATENAME))
*# statesshp@data$STATENAME <- gsub("New Hamphsire", "New Hampshire", as.character(statesshp@data$STATENAME))*
statesshp <- statesshp[**order**(**toupper**(**as.character**(statesshp**@**data**$**STATENAME))),]
suicidedata <- suicidedata[**order**(suicidedata**$**year, **toupper**(**as.character**(suicidedata**$**NAME))),]

**stopifnot**(suicidedata[1**:nrow**(statesshp),]**$**NAME **==** statesshp**@**data**$**STATENAME)

statesshp.adj <- **file.path**(**getwd**(), "statesshp.graph") *# IMO file.path is simpler to create full path than using paste/paste0*
statesshp_nb <- **poly2nb**(statesshp) *# neighborhood*
**nb2INLA**(statesshp.adj, statesshp_nb) *# convert nb to INLA compatible graph*
*## H <- inla.read.graph(filename=statesshp.adj)*
*## print(image(inla.graph2matrix(H), xlab="", ylab=""))*

states1 <- suicidedata**$**states

formula.par <- y **~** 1 **+**
 **f**(states, model="bym", graph=statesshp.adj, constr=TRUE) **+**
 **f**(states1, year, model="iid", constr=TRUE) **+**
 year
model.par <- **inla**(formula.par, family="poisson", data=suicidedata, E=E,
 control.predictor=**list**(compute=TRUE),
 control.compute=**list**(dic=TRUE,cpo=TRUE))

**round**(model.par**$**summary.fixed,3)
*## mean sd 0.025quant 0.5quant 0.975quant mode kld*
*## (Intercept) -0.055 0.014 -0.084 -0.055 -0.027 -0.055 0*
*## year -0.013 0.004 -0.021 -0.013 -0.004 -0.013 0*

m <- model.par**$**marginals.random[[1]][1**:nrow**(statesshp)]
zeta.ST1 <- xi.raw <- **unlist**(**lapply**(m,**function**(x)**inla.emarginal**(exp,x)))

SMR.cutoff<- **c**(0.0, 0.9, 1.0, 1.1,3)
xi.factor <- **cut**(zeta.ST1,breaks=SMR.cutoff,include.lowest=TRUE)
m <- int.raw <- model.par**$**summary.random[[2]][1**:nrow**(statesshp),2]
int.cut <- **c**(**-**0.008,**-**0.001,0,0.001,0.006)
int.factor <- **cut**(m,breaks=int.cut,include.lowest=TRUE)

data.states <- **attr**(statesshp, "data")
**attr**(statesshp, "data")=**data.frame**(data.states, xi_raw = xi.raw, xi_factor=xi.factor)


zeta_df <- model.par**$**summary.random[[1]][1**:nrow**(statesshp),]
**colnames**(zeta_df) <- **sprintf**("%s_zeta", **colnames**(zeta_df))
delta_df <- model.par**$**summary.random[[2]][1**:nrow**(statesshp),]
**colnames**(delta_df) <- **sprintf**("%s_delta", **colnames**(delta_df))
**attr**(statesshp, "data") <- **cbind**(**attr**(statesshp, "data"), zeta_df, delta_df)


**writeOGR**(statesshp, "output", "statesshp_with_inla_generated_data",
 driver="ESRI Shapefile", overwrite_layer=TRUE)

**References**

1. National Cancer Institute. *Joinpoint Regression Program.* Surveillance Research Program, Statistical Methodology and Applications Branch; 2018.

2. Kim H-J, Fay MP, Feuer EJ, Midthune DN. Permutation tests for joinpoint regression with applications to cancer rates. *Statistics in Medicine*. 2000;19(3):335-351. doi:10.1002/(SICI)1097-0258(20000215)19:3<335::AID-SIM336>3.0.CO;2-Z

3. Martínez-Beneito MA, Botella-Rocamora P. *Disease Mapping: From Foundations to Multidimensional Modeling*. CRC Press; 2019.

4. Blangiardo M, Cameletti M. *Spatial and Spatio-Temporal Bayesian Models with R-INLA.* John Wiley & Sons; 2015.

5. Van den Broeck J, Brestoff J, Kaulfuss C. Statistical estimation. In: *Epidemiology: Principles and Practical Guidelines.* Springer Netherlands; 2013:417-438.

6. Knorr-Held L, Besag J. Modeling risk from a disease in time and space. *Statistics in Medicine*. 1998;17(18):2045-2060.

7. Bakka H, Rue H, Fuglstad G-A, et al. Spatial modelling with R-INLA: A review. Published online February 18, 2018. http://arxiv.org/pdf/1802.06350

8. Knorr-Held L. Bayesian modeling of inseparable space-time variation in disease risk. *Statistics in Medicine*. 2000;19(17/18):2555-2567.
