# Supplementary material for: Fatal self-injury in the United States, 1999–2018: Unmasking a national mental health crisis
Source: eClinicalMedicine. 2021 Feb 8;32:100741. doi: 10.1016/j.eclinm.2021.100741 (PMC7910714; doi:10.1016/j.eclinm.2021.100741)
Supplement: Supplementary file 2 [file mmc2.docx]

Summary of spacetime, space only, and no space/spacetime models

| **Summary of Bayesian Space time model [Figs. 5a & b]**  Call:  c("inla(formula = formula.par, data = suicidedata, E = E, control.compute = list(dic = TRUE, ", " cpo = TRUE), control.predictor = list(compute = TRUE))")  Time used:   \| Pre-processing \| Running inla \| Post-processing \| Total \| \| --- \| --- \| --- \| --- \| \| 4.4979 \| 1.6551 \| 0.6345 \| 6.7875 \|   Fixed effects:   \|  \| mean \| sd \| 0.025quant \| 0.5quant \| 0.975quant \| mode \| kld \| \| --- \| --- \| --- \| --- \| --- \| --- \| --- \| --- \| \| (Intercept) \| 44.1714 \| 30.2658 \| -15.1981 \| 44.1528 \| 103.5930 \| 44.1176 \| 0 \| \| year \| 228.9169 \| 10.7950 \| 207.7042 \| 228.9230 \| 250.0782 \| 228.9363 \| 0 \|   Random effects:  Name Model  states BYM model  states1 IID model  Model hyperparameters:   \|  \| mean \| sd \| 0.025quant \| 0.5quant \| 0.975quant \| mode \| \| --- \| --- \| --- \| --- \| --- \| --- \| --- \| \| Precision for the Gaussian observations \| 0.00 \| 0.00 \| 0.00 \| 0.00 \| 0.00 \| 0.00 \| \| Precision for states (iid component) \| 53.60 \| 17.19 \| 29.54 \| 50.32 \| 96.05 \| 44.39 \| \| Precision for states (spatial component) \| 1512.91 \| 308.68 \| 984.34 \| 1485.03 \| 2212.94 \| 1433.85 \| \| Precision for states1 \| 0.00 \| 0.00 \| 0.00 \| 0.00 \| 0.00 \| 0.00 \|   Expected number of effective parameters(std dev): 51.11(0.1152)  Number of equivalent replicates : 3.991  **Deviance Information Criterion (DIC) : 2758.97**  Deviance Information Criterion (DIC, saturated) : 271.83  Effective number of parameters : 54.15  Marginal log-Likelihood: -1509.24  CPO and PIT are computed  Posterior marginals for linear predictor and fitted values computed |
| --- | --- | --- | --- | --- | --- | --- | --- | --- | --- | --- | --- | --- | --- | --- | --- | --- | --- | --- | --- | --- | --- | --- | --- | --- | --- | --- | --- | --- | --- | --- | --- | --- | --- | --- | --- | --- | --- | --- | --- | --- | --- | --- | --- | --- | --- | --- | --- | --- | --- | --- | --- | --- | --- | --- | --- | --- | --- | --- | --- | --- | --- | --- | --- | --- | --- | --- | --- |

| Model with only space (no spacetime)  summary(model.par)  Call:  c("inla(formula = formula.par, data = suicidedata, E = E, control.compute = list(dic = TRUE, ", " cpo = TRUE), control.predictor = list(compute = TRUE))")  Time used:  Pre-processing Running inla Post-processing Total  3.9513 1.2439 0.5013 5.6965  Fixed effects:  mean sd 0.025quant 0.5quant 0.975quant mode kld  (Intercept) 624.6612 48.128 530.1552 624.6616 719.0684 624.6665 0  Random effects:  Name Model  states BYM model  Model hyperparameters:  mean sd 0.025quant 0.5quant 0.975quant mode  Precision for the Gaussian observations 0.00 0.00 0.00 0.00 0.00 0.00  Precision for states (iid component) 5846.35 15026.71 348.92 2300.15 33242.97 779.37  Precision for states (spatial component) 62061.71 1121154.19 290.43 3896.55 350610.17 572.44  Expected number of effective parameters(std dev): 1.915(0.0119)  Number of equivalent replicates : 106.53  **Deviance Information Criterion (DIC) ...............: 3272.57**  Deviance Information Criterion (DIC, saturated) ....: 272.48  Effective number of parameters .....................: -6.222  Marginal log-Likelihood: -1634.54  CPO and PIT are computed  Posterior marginals for linear predictor and fitted values computed |
| --- |

| Model w/no space or no spacetime  Call:  c("inla(formula = formula.par, data = suicidedata, E = E, control.compute = list(dic = TRUE, ", " cpo = TRUE), control.predictor = list(compute = TRUE))")  Time used:  Pre-processing Running inla Post-processing Total  7.3014 1.4693 0.6633 9.4340  Fixed effects:  mean sd 0.025quant 0.5quant 0.975quant mode kld  (Intercept) 614.9786 53.5645 509.6859 614.9766 720.1911 614.9772 0  The model has no random effects  Model hyperparameters:  mean sd 0.025quant 0.5quant 0.975quant mode  Precision for the Gaussian observations 0.00 0.00 0.00 0.00 0.00 0.00  Expected number of effective parameters(std dev): 1.00(1e-04)  Number of equivalent replicates : 204.00  **Deviance Information Criterion (DIC) ...............: 3279.67**  Deviance Information Criterion (DIC, saturated) ....: 211.77  Effective number of parameters .....................: 2.246  Marginal log-Likelihood: -1657.80  CPO and PIT are computed  Posterior marginals for linear predictor and fitted values computed |
| --- |
